# Gravity-Vector Induces Mechanical Remodeling of rMSCs *via* Combined Substrate Stiffness and Orientation

**DOI:** 10.3389/fbioe.2021.724101

**Published:** 2022-02-07

**Authors:** Chen Zhang, Dongyuan Lü, Fan Zhang, Yi Wu, Lu Zheng, Xiaoyu Zhang, Zhan Li, Shujin Sun, Mian Long

**Affiliations:** ^1^ Center for Biomechanics and Bioengineering, Key Laboratory of Microgravity (National Microgravity Laboratory) and Beijing Key Laboratory of Engineered Construction and Mechanobiology, Institute of Mechanics, Chinese Academy of Sciences, Beijing, China; ^2^ School of Engineering Science, University of Chinese Academy of Sciences, Beijing, China

**Keywords:** substrate stiffness, orientation, mechanosensing, nucleus translocation, cytoskeletal remodeling, focal adhesion complex reorganization, cellular morphology

## Abstract

Distinct physical factors originating from the cellular microenvironment are crucial to the biological homeostasis of stem cells. While substrate stiffness and orientation are known to regulate the mechanical remodeling and fate decision of mesenchymal stem cells (MSCs) separately, it remains unclear how the two factors are combined to manipulate their mechanical stability under gravity vector. Here we quantified these combined effects by placing rat MSCs onto stiffness-varied poly-dimethylsiloxane (PDMS) substrates in upward (180°), downward (0°), or edge-on (90°) orientation. Compared with those values onto glass coverslip, the nuclear longitudinal translocation, due to the density difference between the nucleus and the cytosol, was found to be lower at 0° for 24 h and higher at 90° for 24 and 72 h onto 2.5 MPa PDMS substrate. At 0°, the cell was mechanically supported by remarkably reduced actin and dramatically enhanced vimentin expression. At 90°, both enhanced actin and vimentin expression worked cooperatively to maintain cell stability. Specifically, perinuclear actin stress fibers with a large number, low anisotropy, and visible perinuclear vimentin cords were formed onto 2.5 MPa PDMS at 90° for 72 h, supporting the orientation difference in nuclear translocation and global cytoskeleton expression. This orientation dependence tended to disappear onto softer PDMS, presenting distinctive features in nuclear translocation and cytoskeletal structures. Moreover, cellular morphology and focal adhesion were mainly affected by substrate stiffness, yielding a time course of increased spreading area at 24 h but decreased area at 72 h with a decrease of stiffness. Mechanistically, the cell tended to be stabilized onto these PDMS substrates *via* β1 integrin–focal adhesion complexes–actin mechanosensitive axis. These results provided an insight in understanding the combination of substrate stiffness and orientation in defining the mechanical stability of rMSCs.

## Introduction

The stem cell niche, defined as the surrounding microenvironment of both the neighboring cells and the extracellular matrix (ECM), provides biochemical and biomechanical signals to regulate stem cell self-renewal and fate commitment (Lemischka and Moore, 2003; Moore Lemischka, 2006; Mitsiadis et al., 2007; Aichinger et al., 2012; Kordes and Haeussinger, 2013; Turksen, 2015). Physical or mechanical factors (ECM stiffness, mechanical force, topography, cell shape or colony sizes, and others) play a considerably important role in these processes (Discher et al., 2009; Li et al., 2011). It is well known that matrix stiffness directs stem cell fate specification, as it was seen that mesenchymal stem cells (MSCs) can differentiate into osteoblasts, myoblasts, and neurons on a substrate that mimics bone, muscle, and neural stiffness, respectively (Engler et al., 2006; Rowlands et al., 2008; Tse and Engler, 2011). Hereinto mechanosensing is initiated from the varied cell–ECM traction force induced by different substrate stiffness levels, which then alters the intracellular prestress and stem cell or nucleus stiffness and results in the mechanical remodeling of stem cells on their niches (Chowdhury et al., 2010; Swift et al., 2013; Buxboim et al., 2014; Harada et al., 2014). Evidently, these *in vitro* studies, by mimicking or replicating *in vivo* ECM stiffness on a planar substrate, open a window from a biomechanical or biophysical viewpoint for the mechanical remodeling of various types of stem cells.

Substrate orientation also regulates the mechanical remodeling of the cells. These studies are usually designed to elucidate how the gravity vector manipulates the gravisensing mechanisms for a plant or mammalian cell (Vassy et al., 2001; Morita, 2010)—for example, the spreading and mitosis of Chinese hamster ovary cells are sensitive to the change in gravity vector, and randomizing the direction of the gravity has no effects on the division orientation of the point-attached cell in a vertical plane (Helmstetter, 1997). Directed nucleolus sedimentation inside a *Xenopus* oocyte is dominant over thermal fluctuation, implying that the sedimentation of a relatively dense nucleus could initiate cell gravisensing (Feric and Brangwynne et al., 2013). While these static models of orientation change are helpful in elucidating cell mechanosensing, it is still unclear in this process what a role the extracellular microenvironment, such as ECM stiffness, plays and how it is correlated with substrate orientation alteration.

Mechanotransduction is crucial to understand the above-mentioned mechanosensing process. On one hand, substrate stiffness is well sensed by membrane-anchored integrin molecules. F-actin binds to matrix proteins at the focal plane *via* integrin-anchored focal adhesion complexes (FACs) as well as to myosin II elements inside the cell, which initiates extra-intracellular mechanotransduction (Giannone et al., 2007; Irianto et al., 2016). Meanwhile, there are substantial physical links between the nucleoskeleton and cytosolic actin, intermediate filament, or microtubule components (Dahl et al., 2004; Lammerding et al., 2004; Lammerding et al., 2006; Dahl et al., 2008) *via* the linker of nucleus and cytoskeleton (LINC) complex (Crisp et al., 2006; Dahl et al., 2008). These signaling pathways result in a major mechanotransduction axis from the ECM to the nucleus through cytoskeletal (CSK) remodeling. On the other hand, the gravitational force acting on or lost from the organelles is sensed by the cytoskeletons. Loss of gravity alters prestress in the cytoskeleton and is transmitted to the mechanosensitive structures of actin, intermediate filament, and microtubule (Vassy et al., 2001; Bershadsky et al., 2006; Meloni et al., 2011), suggesting that the CSK network could serve as the preferential candidate for intracellular gravisensing. This mechanical signaling can be transmitted though the interactions between F-actin and FACs and induces FAC remodeling to lead the cell adhered stably on substrate (Humphries et al., 2007), which is finally transmitted to the nucleoskeleton *via* LINC. Thus, both the substrate stiffness and orientation likely share the common mechanotransductive pathways, which is required to be identified in a combined approach.

Previously, we quantified how substrate stiffness and microtopography cooperatively direct the differentiation of rMSCs (Li et al., 2013) and maintain the stemness of mouse embryonic stem cells (Lü et al., 2014), suggesting that the CSK remodeling is one of key factors in these processes. Recently, we also elucidated how the substrate orientation affects the mechanical stability of an osteoblast-like cell, where the nucleus translocation due to density difference is mechanically supported by CSK remodeling and FAC reorganization (Zhang et al., 2017). Here we combined the substrate stiffness with substrate orientation mainly because the former is biologically relevant and the latter is a well-defined *in vitro* model. rMSC remodeling was systematically tested for stiffness-varied substrates in three orientations, and the related intracellular events were analyzed for biological homeostasis of the cells.

## Materials and Methods

### Ethics Statement

All experiments involving the use of live animals were conducted in accordance with the NIH Guide for Care and Use of Laboratory Animals, and all the protocols were approved by the CULA at the Institute of Mechanics, Chinese Academy of Sciences.

### Cells and Reagents

Rat bone marrow-derived stem cells (rMSCs) were isolated from 3- to 4-week-old male Sprague–Dawley rats as described previously (Li et al., 2013). Briefly, the rats were sacrificed by cervical dislocation, and the femurs and tibias were then collected. The marrow was flushed out and blown into single cells by an injector, and the collected cells were added into L-DMEM medium (GE Healthcare Life Sciences, Logan, UT, United States) supplemented with 10% fetal bovine serum (Thermo Fisher Scientific, Waltham, MA, United States) and 1 ng/ml bFGF (R&D systems, Minneapolis, MN, United States). The cells were then maintained in a humidified incubator with 95% air/5% CO_2_, 37°C, by refreshing the medium every 3 days. When grown up to 85–90% confluence, the cells were detached using 0.25% trypsin-EDTA for 1 min and passaged into T-25 flask by 1:3 ratio. This procedure was repeated three or four times to collect rMSCs with ∼95% purity.

For CSK staining, FITC-conjugated phalloidin was from Enzo Life Science (Enzo Biochem, Farmingdale, NY, United States), anti-vimentin (Alexa Flour 647-conjugated) and anti-α-tubulin (Alexa Flour 555-conjugated) rabbit monoclonal antibodies (mAbs) were from Cell Signaling Technology (Danvers, MA, United States). For FAC staining, anti-vinculin rabbit mAbs and donkey-anti-rabbit anti-IgG secondary polyclonal antibodies (DyLight 594-conjugated) were from Abcam (Cambridge, Cambridgeshire, UK). For β1 integrin staining, anti-β1 integrin mouse mAbs was from Santa Cruz (Dallas, TX, United States), and the goat-anti-mouse anti-IgG secondary polyclonal antibodies (Alexa Fluor 594-conjugated) were from Abcam. Hochest 33342 for nucleus stain was from Invitrogen (Thermo Fisher Scientific, Waltham, MA, United States). Goat-anti-rat anti-CD11b, CD34, CD45, or CD90 mAbs were purchased from Santa Cruz (United States) and used as biomarkers for identifying the rMSCs as described previously (Li et al., 2013).

### Fabricating PDMS Substrate

Poly-dimethylsiloxane (PDMS) was used to construct the soft gel onto the glass surface *via* soft contact lithography technique (Whitesides et al., 2001; Cheng and Guo, 2004). Briefly, Sylgard 184 (Dow Corning, United States) was used, and two components of a “base” and a “curing” agent were mixed in 10:1, 33:1, or 70:1 ratio (v/v). The mixture was then poured uniformly on the top of glass coverslip. After additional degassing for 12 h, the PDMS was cured at 65°C for 3 h and at room temperature (∼25°C) for 12 h. The cured PDMS substrate so obtained was then adhered onto the pre-processed, dustless coverslip (Corning, Corning, NY, United States) and treated with O_2_ plasma for 1 min to make the surface of the substrate hydrophilic. In the current work, a planar PDMS substrate was used, with a stiffness of 2.5, 0.56, or 0.005 MPa (Wang, 2011; Yang et al., 2011; Sun et al., 2014), while the glass coverslip for tissue culture was used as control, with a stiffness of ∼70 GPa, only for normalizing those data obtained from PDMS substrates with different stiffness levels.

### Substrate-Oriented Cell Culture

**FIGURE 1:**
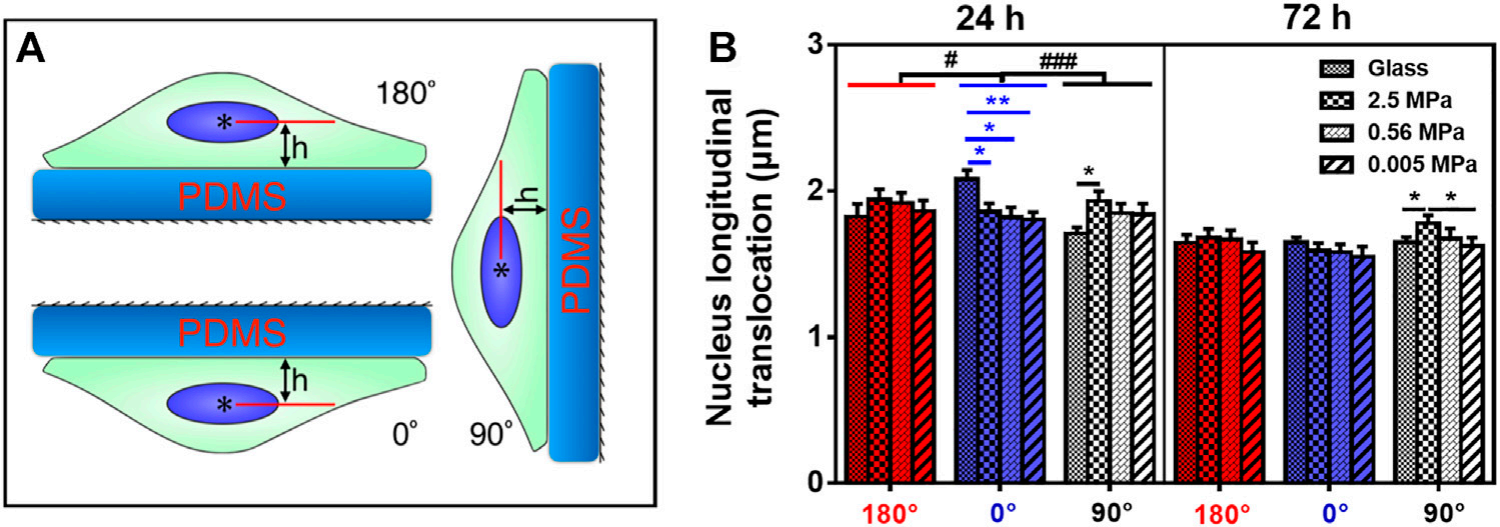
Nucleus translocation of rat bone marrow-derived stem cells (rMSCs) on different stiffness in three oriented substrates. **(A)** Schematic of rMSCs placed onto poly-dimethylsiloxane (PDMS) substrate at 180°, 0°, or 90°. The angle is defined as the one between the outer normal vector of the substrate and the gravity vector in respective orientation, and *h* is the distance between the nucleus centroid ( * ) to the surface of the substrate. **(B)** Nucleus longitudinal translocation of rMSCs onto oriented glass or PDMS substrate with three stiffness substrates. Data were presented as mean ± SE of ∼45 cells from three repeated experiments at 24 or 72 h. ^*^ or ^**^, *t*-test, *p* < 0.05 or 0.01; ^#^ or ^###^, two-way ANOVA test for different groups with different stiffness substrates with cell orientation, *p* < 0.05 or 0.001.

Like those methods described previously (Li et al., 2010; Zhang et al., 2017), rMSCs were placed on three oriented glass coverslips in the presence or absence of PDMS cushion with different stiffness levels (∼2 mm thickness) on the top. Briefly, the cells were seeded on either sterile glass or stiffness-varied PDMS substrate pre-coated with collagen I at 4 μg/cm^2^ overnight. After 24-h pre-growth for steady cell adherence and spread, the substrate was transferred to a custom-made holder and orientated at 180°, 0°, or 90°, respectively (the angle between the substrate outer normal vector and the gravity vector; Figure 1A) for additional 24- or 72-h culture. To reach a similar confluence with the majority of isolated cells at given durations, the cells were seeded at a density of 1 × 10^2^ or 3 × 10^2^ cells/cm^2^ for an additional 72 or 24 h. The effect of hydrostatic pressure among the three orientations was minimized and negligible due to the delicate protocol (Li et al., 2010; Zhang et al., 2017). Triplicate repeats were conducted in each orientation.

### Nuclear and CSK Staining and Parameter Estimation

Morphological change and cytoskeletal expression were determined by immuno-cytochemistry. At given durations, cells grown on oriented glass or PDMS were rinsed with phosphate-buffered saline (PBS), fixed into 4% paraformaldehyde within 1 min for 15 min, and washed and permeated with 0.1% Triton 100-X for 10 min at room temperature. The collected cells were incubated in 1% bovine serum albumin/PBS for 60 min at 37°C to block non-specific staining. Filamentous actin, vimentin, and tubulin were stained with a mixture of phalloidin at 5 μg/ml, anti-vimentin mAbs at 1:800, and anti-tubulin mAbs at 1:50 for 60 min at 37°C. The cells were then washed and incubated with Hoechst 33342 for 10 min to stain the nuclei. Images of the stained cells were examined using a laser confocal microscope (LSM 710, Carl Zeiss AG, Oberkochen, Germany) with a 63× oil-immersion objective at a slicing height of 0.65 μm in a stepwise interval of 0.322 μm for three-dimensional (3D) imaging. Triplicate repeats were done in each case.

Several parameters were obtained from these images: 1) Nucleus translocation, defined as the longitudinal distance between the nucleus centroid and the substrate, was determined by Imaris software (Bitplane, Zurich, Switzerland) through the 3D reconstructed images of stained actin and nucleus; 2) CSK expression was quantified as the mean relative fluorescence intensity (arbitrary unit or AU) of stained actin, vimentin, or tubulin using ImageJ software (National Institutes of Health, Bethesda, MD, United States). To compare these values in distinct orientations in repeated experiments, a calibration curve was built at systematically varied laser power and PMT gain for the same fluorescent probes. To further test the expression difference between the two types of substrates, a ratio of each value onto PDMS to that onto glass was calculated in all the cases. Perinuclear actin stress fibers and vimentin cords, defined previously (Zhang et al., 2017), were used for quantitative analysis; 3) Anisotropy of perinuclear actin, defined in a recent protocol (Boudaoud et al., 2014), was also adopted to quantify the behavior of this type of fibrillar structures. Here a residual eigenvalue, *q*, calculated from the pixel intensity array of a given region of interest, denotes a completely isotropic fibril structure when *q* = 0 or a completely anisotropic fibril structure when *q* = 1. Noticing that the fibrillar isotropy and anisotropy respectively represent the randomized and aligned actin network, this eigenvalue was estimated in the region of the nucleus contour; 4) Cell morphological analysis was simply conducted on the cell contour identified by stained actin. Cell projected area, circularity (= 4π*A*/perimeter^2^), and aspect ratio (= long-axis length/short-axis length) was determined using ImageJ.

### Staining and Functional Blocking of Mechanosensitive Molecules

FAC immunostaining was similar to the procedure detailed above for cytoskeleton staining. Briefly, after washing and fixing, the cells were co-incubated with phalloidin at 5 μg/ml and anti-vinculin mAbs at 1:200 for 60 min at 37°C, rinsed and incubated with secondary antibodies at 1:200 for 60 min at 37°C, and finally incubated with Hoechst 33342 for 10 min. FACs were visualized using confocal microscopy by collecting 0.65-μm-thickness information at the focal plane. The number of total FACs was counted for ∼45 cells in each case, and the area of total FACs was calculated, respectively, using Matlab software. To exclude the potential impact of a different cell size, the resulting FAC number and area for cells were normalized per 1,000 μm^2^ cell area. Similar protocol was applied for β1 integrin immunostaining.

To elucidate the potential mechanotransductive pathways, rMSCs were incubated with anti-β1 integrin, blocking mAbs at 1.5 μg/ml for 1 h per 24 h (Anguiano et al., 2017) in a total of 72 h of culture (Abcam, Cambridge, United Kingdom) or with 50 ng/ml F-actin depolymizer cytochalasin D (Sigma-Aldrich) for 24 h.

### RNA Extraction and qPCR Test

The cultured rMSCs at 72 h were collected on various stiffness levels and orientations. Their total RNA was harvested using RNA extraction kit (Tiangen, Beijing, China) with an in-column DNase digestion step, according to the instructions of the manufacturer. The corresponding cDNA was generated using ReverTra Ace-a (Toyobo, Osaka, Japan) with 1 μg of RNA per reaction in a total volume of 20 μl. A reverse transcriptase-polymerase chain reaction was carried out using GoTaq^®^ qPCR Master Mix with a two-step method as per the user manual (Promega, Madison, WI, United States) and then measured by a quantitative real-time amplification system (QuantStudio 7, Thermo Fisher). The optimized primers for PCR tests are summarized in Supplementary Table S1.

### Statistical Analysis

Three-way ANOVA was performed to test the statistical significance of differences among the three factors of stiffness, orientation, and duration. Two-way ANOVA test, followed by Holm–Sidak test, was used to test the statistical significance of differences in the measured parameters between the two factors of orientation and duration or of orientation and stiffness. For comparisons between any two groups, Student’s *t*-test or Mann–Whitney test was also performed upon data passing the normality test or not.

## Results

### Differential Regulation of Substrate Orientation Onto PDMS Substrate

Different cell types present diverse phenotypes together with varied ECM stiffness in a pericellular microenvironment. Here we first tested nucleus longitudinal translocation in rMSCs onto typical PDMS substrates when glass coverslip served as the control (Figure 1A). The orientation dependence of nucleus translocation for rMSCs onto glass was presented with a higher value at 0° for 24 h (Figure 1B), consistent with that previously described for MC3T3-E1 cells (Zhang et al., 2017). When rMSCs were placed onto 2.5 MPa PDMS, nucleus translocation was relatively higher at 90° for 24 or 72 h (Figure 1B). These data indicated that the orientation dependence of nuclear translocation likely appeared for rMSCs on 2.5 MPa PDMS in a time-dependent manner. We further tested the combined impacts of substrate stiffness and orientation and found that both factors presented a significant difference in regulating nucleus longitudinal translocation at 2.5 MPa PDMS (symbol # in Figure 1B). Specifically, the translocation onto 2.5 MPa PDMS was lower at 0° for 24 h but higher at 90° for 24 and 72 h compared with those onto glass, potentiating the different mechanisms of substrate stiffness in regulating rMSC stability onto an oriented substrate.

It is well known that PDMS and glass substrates have distinctive properties in both elasticity and chemistry. Thus, we added two more PDMS stiffness levels to further test their mechanical remodeling under gravity vector. On either 0.56 or 0.005 MPa PDMS (Figure 1B), the rMSCs presented no orientation dependence of their nucleus translocation for 24 or 72 h, which is different from the above-mentioned observations onto 2.5 MPa PDMS. This was further verified by testing the statistical differences for combined effects of stiffness and orientation. No significant distinctive translocation in three orientations was observed on these softer PDMS (^#^ *p* ≥ 0.408) even with slightly lower values at 0° for 24 or 72 h. Taken together, these results indicated that the orientation dependence of nuclear translocation is also stiffness-dependent and only presented onto stiffer PDMS substrate.

### Global CSK Expressions on PDMS Substrates

**FIGURE 2:**
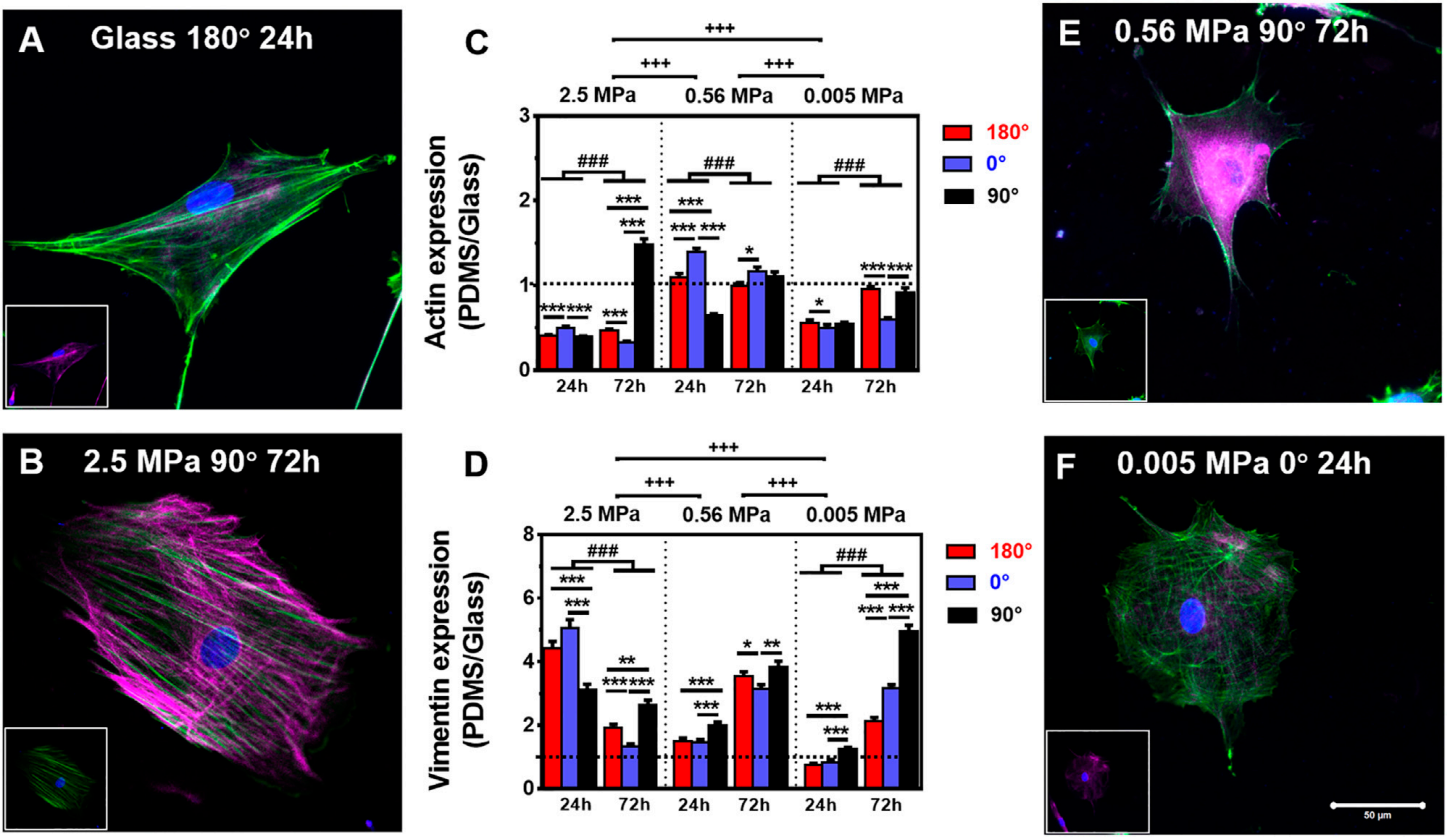
Comparison of actin (green) and vimentin (magenta) expressions. **(A,B,E,F)** Typical merged cytoskeletal images for cells placed onto glass: **(A)** 180°, 24 h, 2.5 MPa, **(B)** 90°, 72 h, 0.56 MPa, **(E)** 90°, 72 h, or 0.005 MPa, **(F)** 0°, 24 h, poly-dimethylsiloxane (PDMS). The insert in each panel denotes the isolated cytoskeletal image for clarity. Bar = 50 µm. **(C,D)** Relative fluorescent intensity onto PDMS normalized to that onto glass was plotted for actin **(E)** or vimentin **(F)** in three orientations and presented as mean ± SE for ∼45 cells from three repeated experiments at 24 or 72 h. ^*^, ^**^, or ^***^, *t*-test, *p* < 0.05, 0.01, or 0.001; ^###^, two-way ANOVA test for different groups with different time with cell orientation, *p* < 0.001; ^+++^, three-way ANOVA test for differences among stiffness, orientation, and duration, *p* < 0.001.

It was indicated previously that the orientation dependence of nuclear translocation is mainly relying on mechanical pathways *via* cytoskeleton remodeling and focal adhesion reorganization (Zhang et al., 2017). Next, we tested how CSK remodeling is associated with differential nucleus longitudinal translocation onto 2.5 MPa PDMS. Compared with high actin expression (Figure 2A) but low vimentin expression (insert in Figure 2A) onto glass, these CSK protein expressions seemed reversed onto 2.5 MPa PDMS, that is, with low actin expression but high vimentin expression at 24 h. Quantitative analyses further supported these observations. Indeed actin expression normalized to the one onto glass was ∼50% reduced onto 2.5 MPa PDMS in all cases, except of the one at 90° for 72 h (Figure 2B and insert for vimentin), where it yielded ∼1.5-fold higher onto 2.5 MPa PDMS (Figure 2C). Intriguingly, the exceptional difference in extremely high actin expression was positively related to the higher nucleus translocation at 90° (dotted bars in Figure 1B). By contrast, vimentin expression is ultimately reversed onto PDMS, that is, 3.0–5.0-fold higher for 24 h or 1.2–2.5-fold higher for 72 h in all cases (Figure 2D). These data indicated that placing rMSCs onto 2.5 MPa PDMS reduced the actin expression but fostered the vimentin expression as compared with those onto glass, implying that the mechanical stability of rMSCs could be achieved relying more on vimentin and less on actin onto 2.5 MPa PDMS or more on actin and less on vimentin onto stiff glass. More importantly, the differential distributions of actin and vimentin onto 2.5 MPa PDMS and glass were consistent with those above-mentioned orientation dependences of nucleus longitudinal translocation. Compared with those onto glass, the low nucleus translocation onto 2.5 MPa PDMS at 0° for 24 h was associated with a relatively low actin and high vimentin expression, suggesting that high vimentin expression at 0° stabilizes the location of the nucleus when actin is reduced significantly by a short duration. By contrast, high nucleus translocation at 90° for 72 h was associated with high actin and high vimentin expressions, indicating that long-duration maintenance at 90° needs more cytoskeletons to resist the nucleus translocation or hold the nucleus steadily (also seen in those perinuclear vimentin distributions below). Additionally, the normalized tubulin expression fluctuated around unity (0.7–1.3) in all cases (Supplementary Figure S1), implying that tubulin contributes much less to this differential mechanism between the two types of substrates. Collectively, the orientation difference of nucleus translocation and global CSK presentation exists onto 2.5 MPa PDMS, with high vimentin expression at 0° for 24 h or high actin and vimentin expressions at 90° for 72 h. Meanwhile, those absolute fluorescence intensities of cytoskeletal protein expression also supported the above-mentioned observations using normalized ones. Onto 2.5 MPa PDMS, low actin, high vimentin, and low tubulin expressions were observed in three orientations for 24 or 72 h, even with a few exceptional cases of high actin expression at 90°, indifferent vimentin expression at 0°, and reversely high tubulin expression at 180° or 0° (Supplementary Figure S2).

We further tested global CSK expressions on softer PDMS substrates. On 0.56 and 0.005 MPa PDMS, CSK protein expressions presented different orientation-dependent patterns from the one on 2.5 MPa (Figures 2E,F and inserts). On 0.56 MPa PDMS, the cells yielded a high actin expression at 0° for 24 or 72 h, all of which were comparable with those on glass except of one case at 180° for 24 h (Figure 2C). By contrast, vimentin expression was relatively high at 90° for 24 or 72 h (Figure 2D) with 3.1–3.8-fold enhancement than those onto glass, consistent with the high nuclear translocation at 90° at long duration (*cf.* Figure 1B) and also implying a compensatory role of vimentin to support nucleus stability. On 0.005 MPa PDMS, the cells presented a relatively low actin expression, especially at 0° for 24 or 72 h, compared to those onto glass (Figure 2C). Vimentin expression was still lowered for 24 h but enhanced for 72 h especially at 90° (Figure 2D).

Finally, we tested typical mechanosensitive gene expressions at 72 h. Data indicated that the expression of actin, vimentin, or α-tubulin was indifferent on varied substrate stiffness and orientation, implying that the cytoskeleton is favored to maintain their gene level in a conservative way on the current settings (Supplementary Figure S3).

### Distinct Distributions of Perinuclear Cytoskeletons Onto PDMS Substrate

**FIGURE 3:**
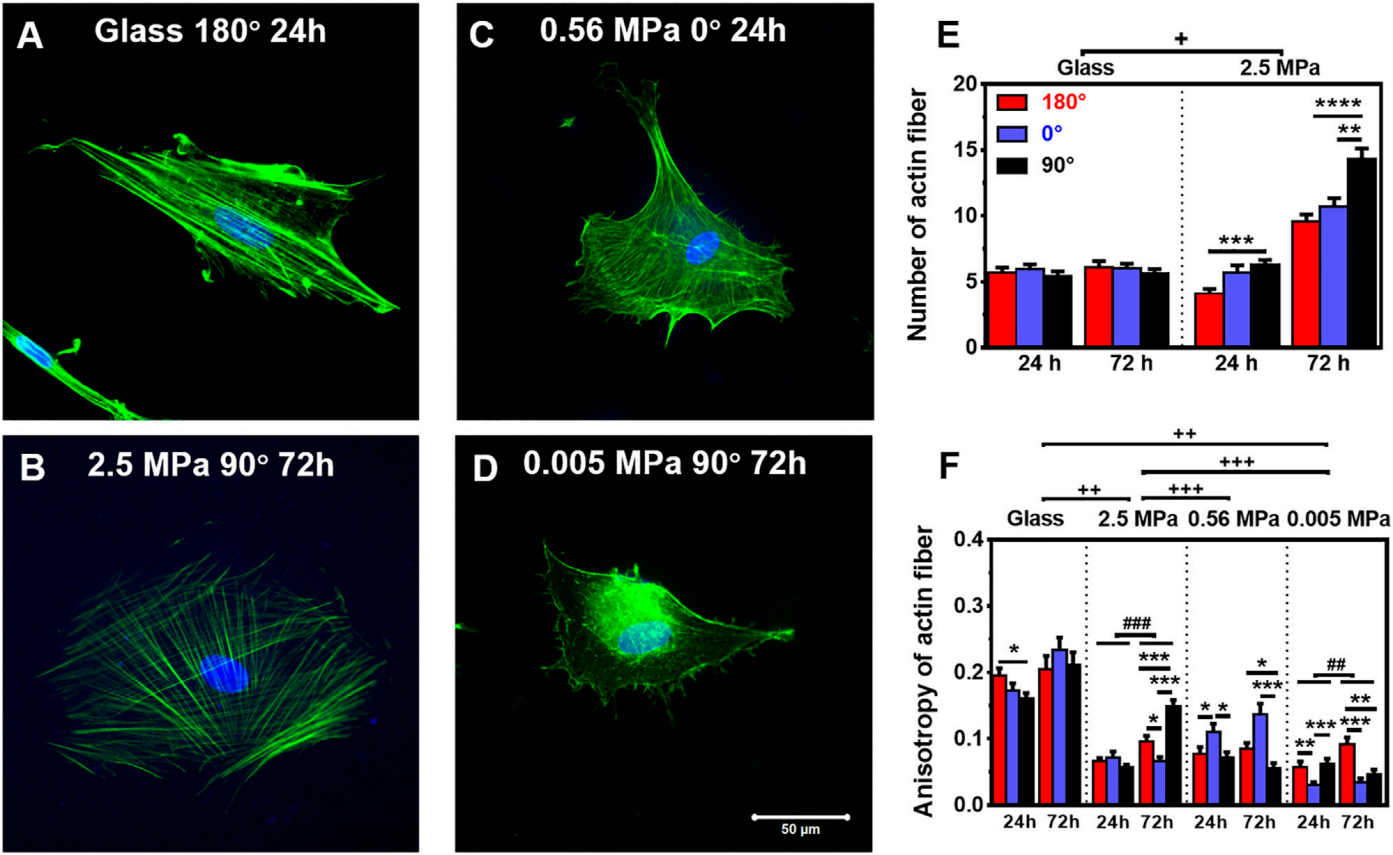
Structure and number of actin stress fibers. **(A–D)** Typical images of perinuclear actin fibers onto glass **(A)** 180°, 24 h, 2.5 MPa, **(B)** 90°, 72 h, 0.56 MPa, **(C)** 0°, 24 h, or 0.005 MPa, **(D)** 90°, 72 h poly-dimethylsiloxane. Bar = 50 µm. **(E,F)** Number **(E)** and anisotropy **(F)** of stress fibers onto the two types of substrates at 24 or 72 h. Here the number was defined previously (Zhang et al., 2017), and the anisotropy term was adopted from Boudaoud *et al*. (2014) (as referred in “Materials and Methods”). Data were presented as mean ± SE for ∼45 cells from three repeated experiments in three orientations. ^*^, ^**^, or ^***^, *t*-test, *p* < 0.05, 0.01, or 0.001; ^##^ or ^###^, two-way ANOVA test for different groups with different time with cell orientation, *p* < 0.01 or 0.001; ^+^, ^++^, or ^+++^, three-way ANOVA test for differences among stiffness, orientation, and duration, *p* < 0.05 0.01 or 0.001.

Not only the global presentation of actin within entire cell but also its localized distribution at the vicinity of the nucleus is crucial in manifesting CSK remodeling and maintaining nucleus stability. We further compared the distribution of perinuclear actin stress fibers onto glass or PDMS substrate. The fibers are likely uniformly aligned with high intensity onto glass (Figure 3A) but randomly oriented with low intensity onto 2.5 MPa (Figure 3B) or even unmeasurable onto 0.56 (Figure 3C) and 0.005 MPa (Figure 3D) PDMS, presenting a significant difference in perinuclear fibers between the two types of substrates. This observation was confirmed by the quantitative analyses that the number of perinuclear fibers was higher onto PDMS than those onto glass for 72 h (Figure 3E). At this duration, the fiber number onto 2.5 MPa PDMS was extremely higher at 90°, further supporting the consistency between global actin expression and nucleus translocation onto PDMS. We also compared the anisotropy of perinuclear fibers on all three PDMS substrates using the residual eigenvalue described previously (Boudaoud et al., 2014). It yielded lower values for cells onto PDMS (∼0.05–0.15) than those onto glass (∼0.15–0.25) in three orientations at two durations (Figure 3F), consistent with the above-mentioned observations of randomized fibers onto PDMS and aligned fibers onto glass from confocal images (Figures 3A,B). We also counted the number of cells with branched perinuclear actin network from the total cells observed. The fractioned number was again increased with time onto either substrate and yielded higher values onto 2.5 MPa PDMS than those onto glass (Table 1), further confirming the occurrence of high randomization or low anisotropy of the actin network for the cells onto PDMS. Similar to those orientation dependences of global actin expression (Figure 2C) and perinuclear fiber presentation (Figure 3E), the number of actin fibers was higher at 90° than that at 180° or 0° onto the same PDMS substrate for 72 h. These results implied that the relatively branched and stronger fibers are favorable in this orientation to maintain the nucleus stability. Taken together, mechanical support for rMSC stability is mostly attributed to the perinuclear stress fibers with a large number, low anisotropy, and low intensity onto PDMS as compared to those with a small number, high anisotropy, and high intensity onto glass. Onto a softer PDMS substrate at 0.56 MPa, a large number of tiny actin filaments (Figure 3C) tended to take over those stress fibers across the nucleus that appeared onto glass, with a relatively high expression and anisotropy but low mechanical support to nucleus translocation (Figure 3F). This substrate seemingly served as a transition one between the 2.5 and 0.005 MPa PDMS substrates since there was no visible filament with quite low anisotropy values onto 0.005 MPa PDMS (Figure 3F).

**TABLE 1:** Fractioned number of rMSCs with branched perinuclear actin fibers.

	Glass			2.5 MPa PDMS		
24	180°	0°	90°	180°	0°	90°
	3/45	2/45	2/45	6/46	10/45	13/45
72	3/45	5/45	3/45	24/45	25/45	36/45

**FIGURE 4:**
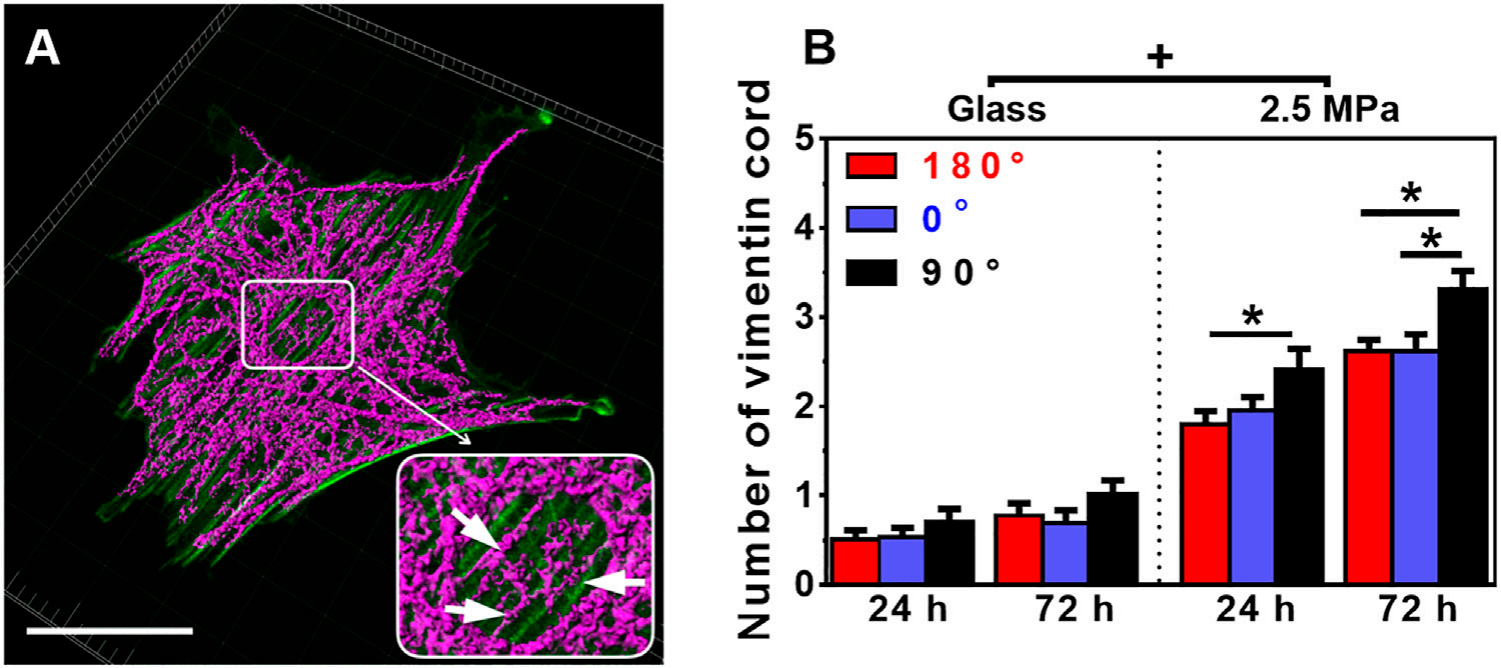
Structure and number of vimentin cords. **(A)** Typical images of perinuclear vimentin cords (arrows in the insert) onto poly-dimethylsiloxane (PDMS) substrate (0°, 72 h). Bar = 50 µm. **(B)** The number of vimentin cords onto 2.5 MPa PDMS and glass, defined previously (Zhang et al., 2017), was compared in three orientations. Data were presented as mean ± SE for 45 cells from three repeated experiments at 24 or 72 h. ^*^, *t*-test, *p* < 0.05; ^+^, three-way ANOVA test, *p* < 0.05.

We also compared the perinuclear vimentin cords onto the two types of substrates. Onto 2.5 MPa PDMS (those images at 0.56 and 0.005 MPa were not able to be reconstructed for quantification), the cords were formed surrounding the nucleus (Figure 4A). The number of vimentin cords yielded higher values at 90° for 24 or 72 h (Figure 4B), partially supporting the orientation dependence of nucleus translocation and global vimentin expression at 90° for 72 h. By contrast, few vimentin cords were visible onto glass (Figure 4B). As indicated, the distribution of vimentin cord number was narrowed down to 0 or 1 onto glass but centered around 2 or 3 onto PDMS (Supplementary Figure S4). Moreover, vimentin onto PDMS tended to be dispersedly distributed from the nucleus to the cell edge, specifically extending into those lamellipodia (Figure 4A) where the actin presentation is low (*cf.*, Figure 2B). These results implied that the vimentin network also provided structural bases for supporting the cell as actin does.

### FAC Reorganization on PDMS Substrate

**FIGURE 5:**
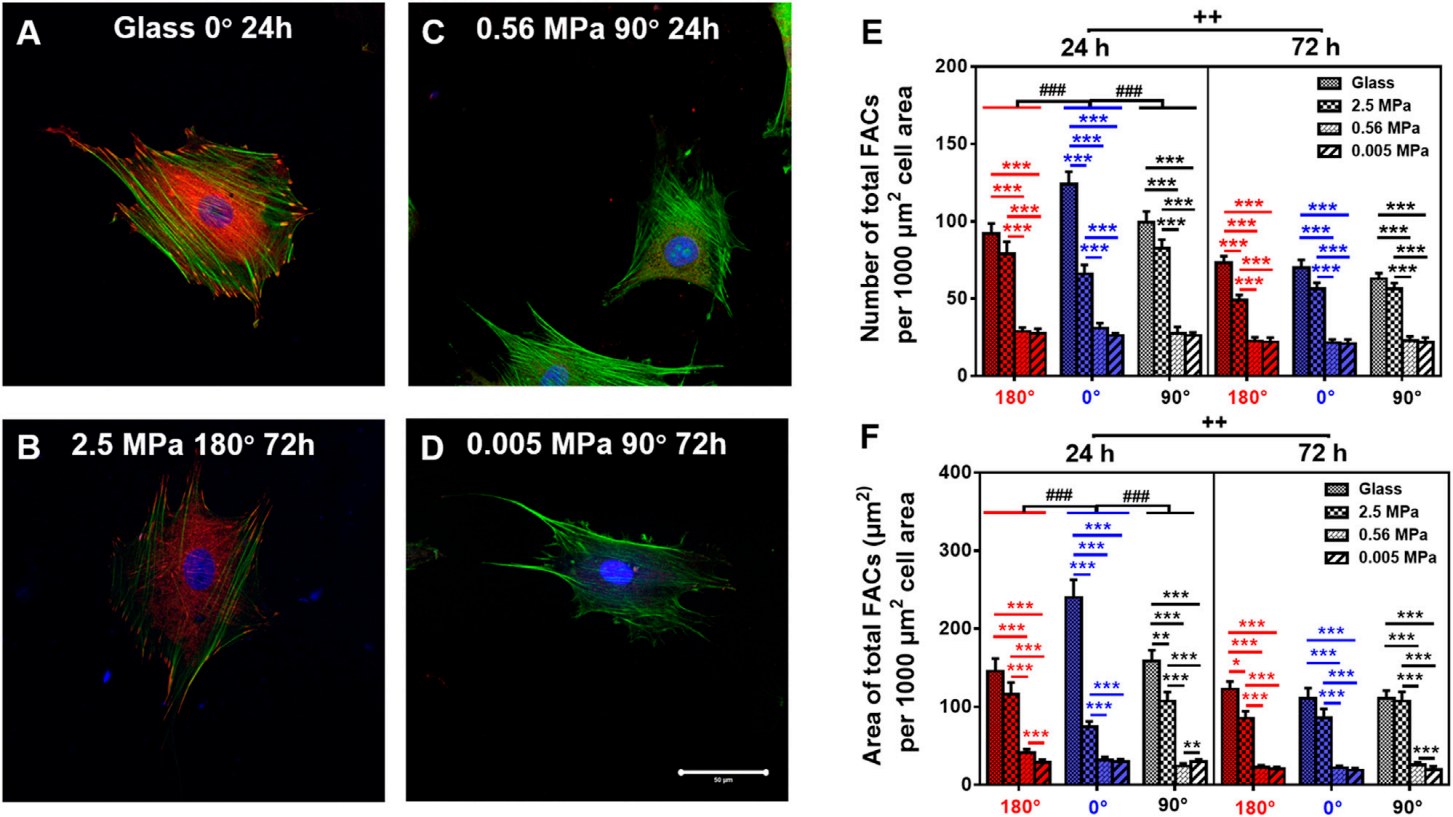
Comparison of focal adhesion complex (FAC) (green, actin; red, vinculin) formation. **(A–D)** Typical images for cells placed onto glass **(A)** 0°, 24 h, 2.5 MPa, **(B)** 180°, 72 h, 0.56 MPa, **(C)**, 90°, 24 h, or 0.005 MPa, **(D)**, 90°, 72 h poly-dimethylsiloxane. Bar = 50 µm. **(E,F)** The number **(E)** and area **(F)** of total FACs (normalized per 1,000 μm^2^ cell area) for cells onto the four different stiffness of substrate in three orientations were presented as mean ± SE of ∼45 cells from three repeated experiments at 24 or 72 h. ^*^, ^**^, or ^***^, *t*-test, *p* < 0.05, 0.01, or 0.001; ^###^, two-way ANOVA test for different group with different time with cell orientation, *p* < 0.001; ^++^, three-way ANOVA test for differences among stiffness, orientation, and duration, *p* < 0.01.

Focal adhesion complex is required to anchor the cell onto the substrate mechanically. Thus, we compared the FAC reorganization onto glass (Figure 5A) or 2.5 (Figure 5B), 0.56 (Figure 5C), or 0.005 (Figure 5D) MPa PDMS in three orientations at two durations. Global differences were again found in normalized number (Figure 5E) and area (Figure 5F) of total FACs between the two types of substrates, presenting lower values onto PDMS (especially on softer substrates with 0.56 and 0.005 MPa) than those onto glass. Meanwhile, both the values of FAC number and area were higher at 0° onto glass for 24 h, consistent with the orientation dependence found for MC3T3-E1 cells (Zhang et al., 2017). By contrast, the value was indifferent onto three PDMS substrates in three orientations at two durations, implying that FAC formation happens in a stiffness-dependent but orientation- and time-insensitive manner onto these relatively soft substrates. This should not be surprising since the mechanical strength of the existing FACs is sufficiently enough to stabilize the cells (Zhang et al., 2017).

### Stable Cell Morphology on the Substrates

**FIGURE 6:**
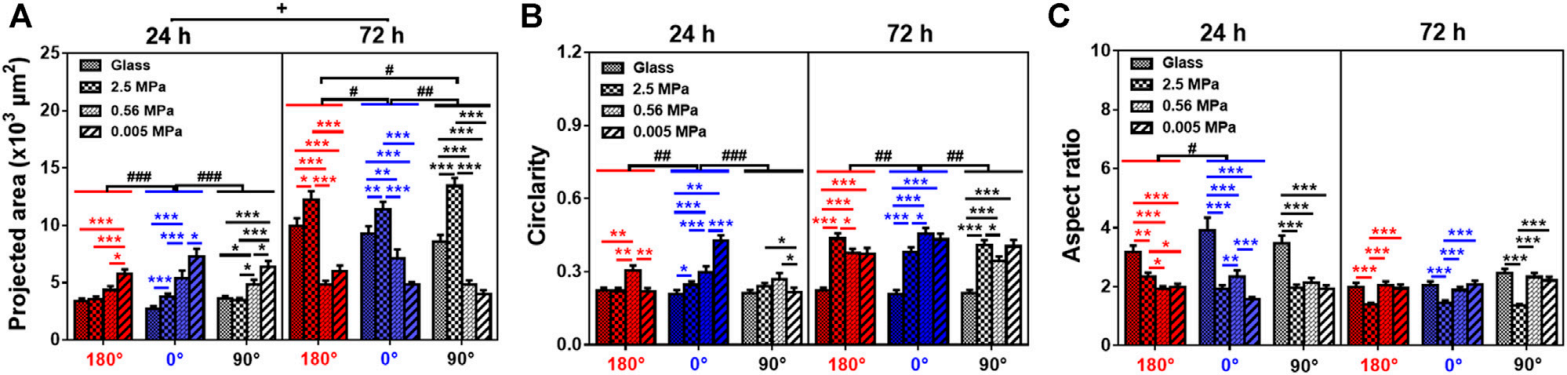
Morphological change of rat bone marrow-derived stem cells (rMSCs). Area **(A)**, circularity **(B)**, and aspect ratio **(C)** of rMSCs onto glass or 2.5, 0.56, or 0.005 MPa poly-dimethylsiloxane in three orientations. Data were collected from those images for cell contour (cf. Supplementary Figure S5) and presented as mean ± SE of ∼45 cells from three repeated experiments at 24 or 72 h. ^*^, ^**^, or ^***^, *t*-test, *p* < 0.05, 0.01, or 0.001; ^#^, ^##^, or ^###^, two-way ANOVA test for different groups with different time with cell orientation, *p* < 0.05, 0.01, or 0.001; ^+^, three-way ANOVA test for differences among stiffness, orientation, and duration, *p* < 0.05.

Lastly, we compared the morphological alterations of rMSCs since the cell is stabilized onto the substrate *via* reorganized FACs. Here the cell contour was identified by actin staining images (Supplementary Figure S5), and the projected area, circularity, and aspect ratio were then determined on three PDMS and glass substrates. Global differences of the three parameters were observed between the two types of substrates or the two durations (Figure 6). At 24 h, there was a striking unanimity in all three orientations, that is, the area gradually increased with decreased stiffness, the circularity onto PDMS was slightly higher at 0° but comparable at 180° or 90°, and the aspect ratio on PDMS was significantly lower in all three orientations. These data indicated that the cells tended to become less long and narrow onto PDMS even of a similar size with those onto glass. At 72 h, both the area and circularity were relatively higher, but the aspect ratio was lower, onto 2.5 MPa PDMS; while the area gradually decreased, the circularity was higher, but the aspect ratio was maintained onto 0.56 and 0.005 MPa PDMS, suggesting that the cells became large in size with a spherical shape at 2.5 MPa but small in size with a spherical shape at 0.56 and 0.005 MPa. It was also noted that the decrease of the area at 72 h onto 0.56 and 0.005 MPa PDMS was correlated to the high vimentin increase (*cf.* Figures 2D, 4B). Additionally, the area and circularity were increased, but the aspect ratio was decreased with time for the cells onto PDMS, similar to the time-dependent morphological alterations onto glass except of the case of time-independent cell circularity. Finally, stiffness dependence and orientation independence were found in cell morphology, consistent with our previous observations for MC3T3-E1 cells (Zhang et al., 2017).

### Regulation of Cellular Mechanotransduction

**FIGURE 7:**
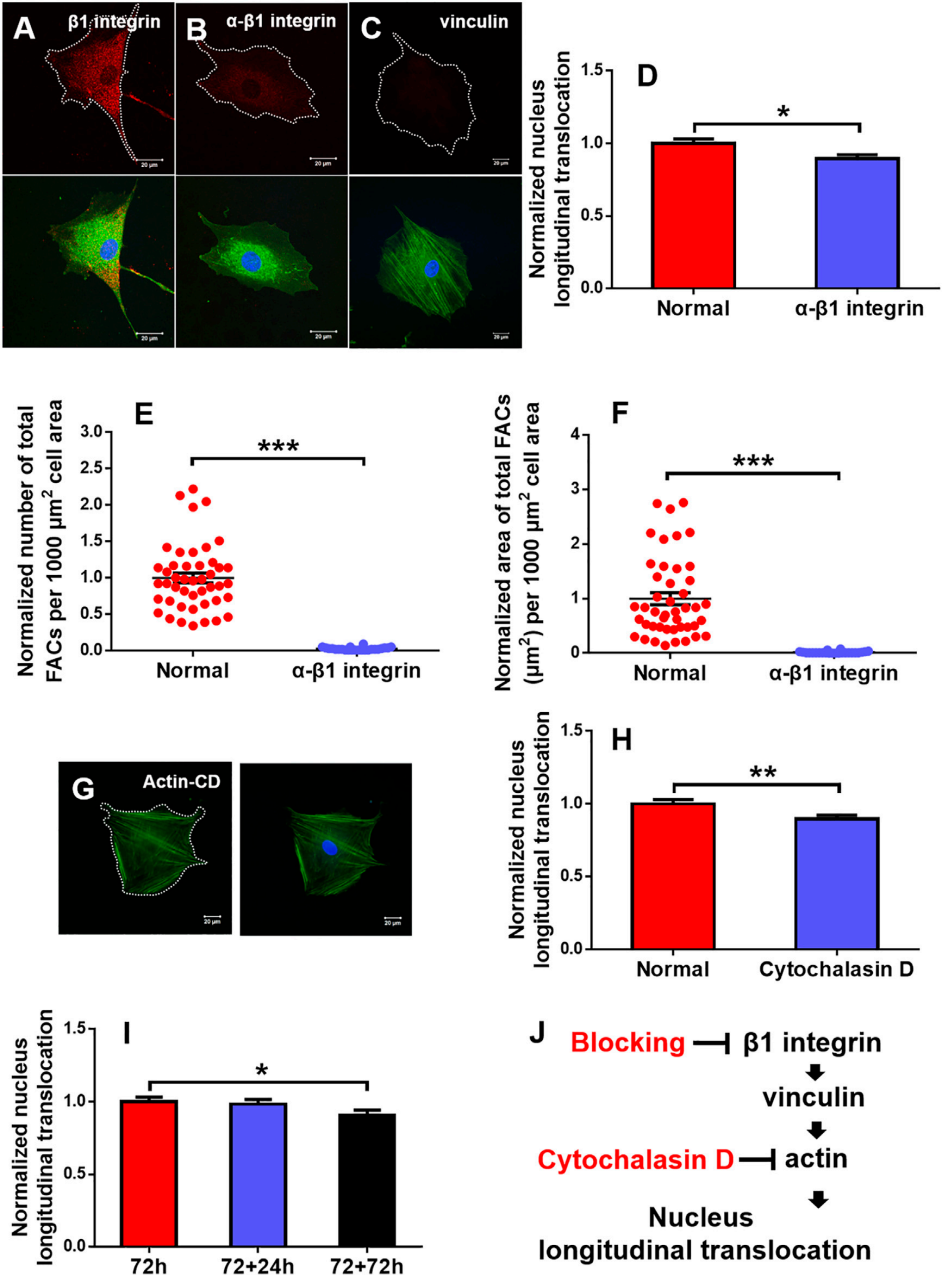
Regulation of mechanotransductive pathways. Typical immunofluorescence staining of β1 integrin **(B)** (red), vinculin **(C)** (red), and actin **(G)** (green; blue, cell nucleus) was presented at 90° on 2.5 MPa poly-dimethylsiloxane (PDMS) for 72 h after β1 integrin function blocking when the one without β1 integrin blocking served as control **(A)** or cytochalasin D disturbed F-actin **(G)**. Bar = 20 µm. The nucleus longitudinal translocation was normalized to control for β1 integrin blocking **(D)** or cytochalasin D disturbed F-actin **(H)** at 90° on 2.5 MPa PDMS for 72 h. The number **(E)** and area **(F)** of total FACs were normalized to their respective controls. Nucleus longitudinal translocation for additional 24 and 72 h at 90° on 2.5 MPa PDMS was normalized to the one for 72 h **(I)**. ^*^, ^**^, or ^***^, *t*-test, *p* < 0.05, 0.01, or 0.001. Schematic of cellular mechanotransductive pathways. Inhibitors of key elements were depicted in red **(J)**. CD, cytochalasin D.

The above-mentioned results indicated that optimizing substrate mechanics and orientation represents a critical step for maintaining the efficient longitudinal translocation of cell nucleus. To elucidate the mechanotransductive pathways involved in affecting rMSC nucleus longitudinal translocation, rMSCs grown typically at 90° on 2.5 MPa PDMS for 72 h were incubated with blocking mAbs against β1 integrin or F-actin de-polymerizer cytochalasin D. The results indicated that β1 integrin expression was significantly decreased, and the fluorescence dots originally existing between rMSCs and the substrate in normal control (Figure 7A) disappeared after blocking (Figure 7B), which is positively correlated with the reduced nucleus longitudinal translocation (Figure 7D). Meanwhile, vinculin expression was lowered (Figure 7C), the number (Figure 7E) and area (Figure 7F) of total FACs were significantly reduced, and actin stress fibers became smaller and thinner (lower panels in Figures 7A–C). Similar observations were found by treating F-actin with cytochalasin D, resulting in remarkably reduced nucleus longitudinal translocation (Figures 7G,H).

To further test if the orientation effect is reversible, the rMSCs originally placed at 90° for 72 h were re-placed horizontally for an additional +24 or +72 h. The results indicated that the nucleus longitudinal translocation was still visualized at +24 h but significantly lower at +72 h, implying that, in addition to substrate stiffness, substrate orientation is also involved in maintaining the nuclear longitudinal translocation of rMSCs and that this orientation effect seemed to be reversible (Figure 7I). Taken together, these results suggested that the typical integrin–FACs–actin mechanotransductive axis plays a key role in regulating rMSC nucleus longitudinal translocation in response to substrate stiffness and orientation (Figure 7J).

## Discussion

**FIGURE 8:**
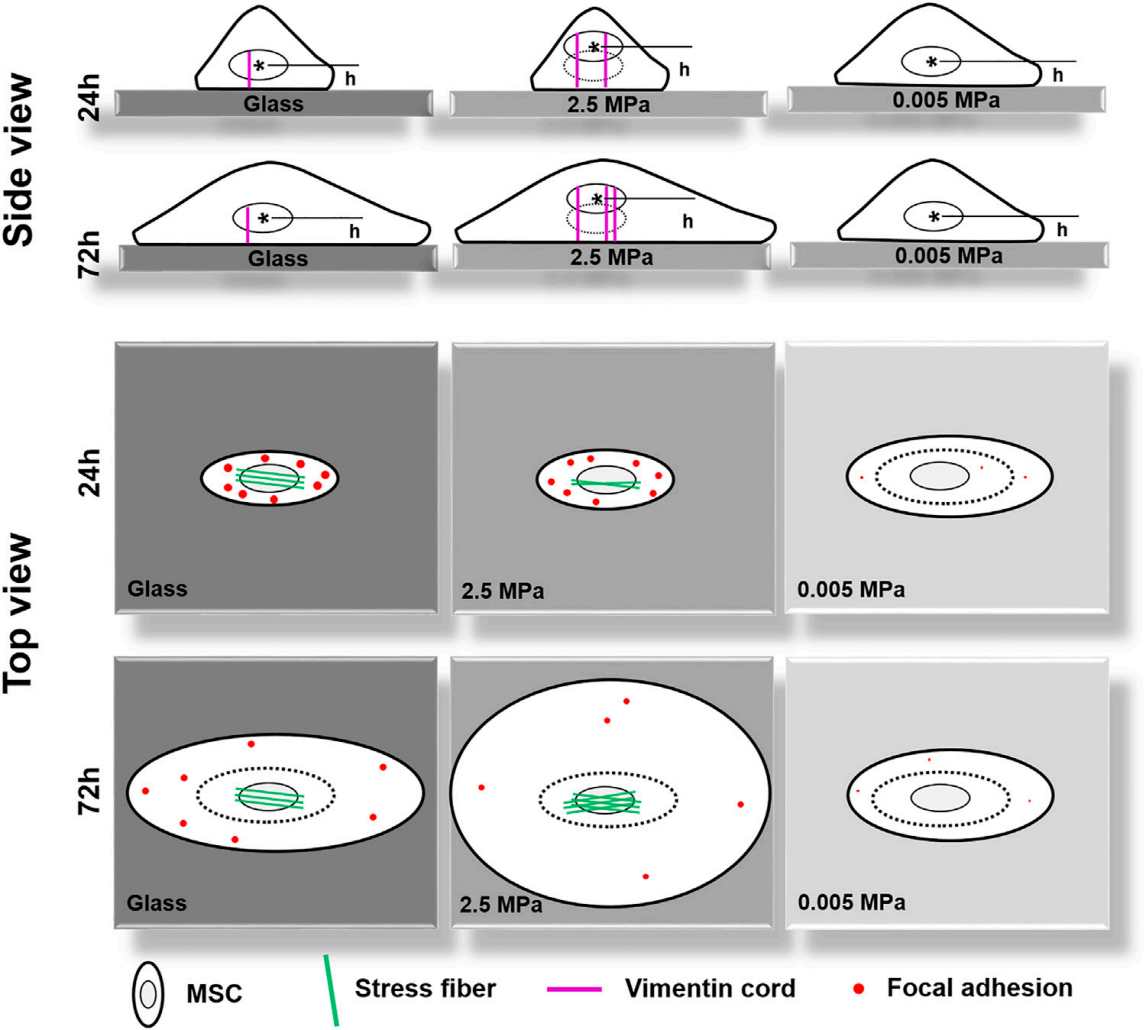
A working model proposed for illustrating the coupled impacts of substrate stiffness and orientation on gravity-vector induced mechanical remodeling of rat bone marrow-derived stem cells. Here are two key points: (1) On stiff poly-dimethylsiloxane (PDMS), the orientation dependence of nuclear longitudinal translocation is similar to that onto glass *via* cytoskeletal remodeling and focal adhesion reorganization; (2) Soft PDMS tends to regulate cell morphology, spreading, and focal adhesion formation without a visible orientation dependence of nuclear translocation. Thick cycle, cell membrane; thin cycle, nucleus (also thin dotted cycle in the right panel); magenta lines, vimentin cords; green lines, actin fibers; red dots, large- or small-sized focal adhesions. *h*, the distance from the nuclear centroid (_*_) to the bottom surface of the substrate.

In the current work, we attempted to elucidate the mechanical remodeling of rMSCs by integrating two biophysical factors of substrate stiffness and orientation. In contrast to those previous works designed for understanding their respective contributions, these combinations initiated the distinct nucleus longitudinal translocation in edge-on orientation at specific durations when the cells were placed onto stiffness-varied PDMS substrates. Not only these differences came from the global expressions of lowered actin and enhanced vimentin over the entire cell region onto PDMS but also they were attributed to the formation of isotropic tiny actin stress fibers on the softer PDMS substrates. Meanwhile, the low number or area of FACs was sufficient to anchor the cell stably onto the PDMS substrates. As a whole, significant differences were found among three PDMS stiffness, not only in nucleus translocation but also in CSK remodeling and FAC reorganization in their respective orientation-dependent patterns. On stiff PDMS, the orientation dependence of nuclear longitudinal translocation is presented *via* cytoskeletal remodeling and focal adhesion reorganization, while the soft PDMS tends to regulate cell morphology, spreading, and focal adhesion formation without visible orientation dependence of nuclear translocation (Figure 8).

Cellular mechanosensing to these combined microenvironments is biologically relevant since these stiffness and orientation are usually coupled together physiologically. *In vivo*, MSCs are required to be activated and transferred from the soft bone marrow to stiff, fibrotic regions for tissue regeneration and repair (Discher et al., 2009). In *in vitro* studies, substrate stiffness serves as a key factor to regulate the fate commitment of MSCs biomechanically (Engler et al., 2006; Discher et al., 2009; Li et al., 2011; Her et al., 2013; Wen et al., 2014; Mao et al., 2016). Meanwhile, the change in substrate orientation is not only associated with gravity vector-directed events but also correlated to mechanically induced cell remodeling in daily life. In fact, a stem cell anchored onto ECM varies its orientation frequently due to the posture change of the human body, and hence substrate orientation alteration *in vitro* could serve as a simple model to elucidate mechanosensing and mechanotransduction in a static state (Helmstetter, 1997; Li et al., 2010; Lü et al., 2014). Thus, the current work not only provided a platform to combine the substrate stiffness and orientation together but also it can be applied to elucidate mechanical remodeling of MSCs onto a physiologically mimicking substrate. A well-defined planar substrate with appropriate stiffness and orientation is crucial to define stem cell behaviors in stem cell biology and mechanotransduction.

Mechanical remodeling of MSCs upon the two combined factors is quite different from those found with their individualized factors. Physical or mechanical clues manipulate stem cell functions by altering the cell shape and re-organizing the cytoskeletal network (Dado et al., 2012). On one hand, a stiff matrix favors MSC proliferation by enhancing the expressions of cell-adhesive molecules to present counterbalancing forces to the substratum. Substrate stiffness also plays a key role in regulating MSC circularity and projected area upon distinct capacity of cell adhesion to a stiff or soft substrate. Moreover, the stiffness determines the fate commitment of MSCs by altering the cell traction force and changing the nuclear translocation of transcript factors (Kilian et al., 2010) or by inducing the specific biomarker expression of differentiated cells cooperatively with the substrate topography and dimension (Li et al., 2013). On the other hand, inversing or tilting the substrate cannot alter the number of attached osteoblasts but vary significantly the cell area and cycle in a time-dependent manner (Mitsiadis et al., 2007; Kordes and Haeussinger, 2013). Meanwhile, orientation-dependent nucleus longitudinal translocation in a MC3T3-E1 cell in varied orientations is well correlated with the remodeling of perinuclear actin stress fibers and vimentin cords and the reorganization of FAC area and size (Zhang et al., 2017). In the current work, these gravity vector-directed orientation dependences were found to be altered. While the obvious nucleus translocation and cytoskeleton remodeling for rMSCs was presented onto 2.5 MPa PDMS, it disappeared for rMSCs onto softer PDMS substrates (Figures 1, 2). These different patterns are presumably attributed to the different mechanically induced cell remodeling on stiffness-varied substrates. As a result, the slightly large, round cell shape was presented onto soft PDMS at long duration, which was supported by comparable FAC number with those onto glass (Figures 5, 6). While these physical or mechanical signals are known to present the differential effects on rMSC functions, the underlying signaling pathways were associated with β1 integrin, FACs, and cytoskeleton (Figure 7).

Moreover, cell remodeling is specific when combining the two mechanical factors. For cytoskeleton remodeling, orientation-specific nucleus translocation at 0° for 24 h or at 90° for 72 h is positively correlated with the differential expressions of actin and vimentin between the two types of substrates (Figures 1, 2). While perinuclear actin tends to form aligned, high-strength stress fibers onto glass that is consistent with those previous observations (Curran et al., 2005; Lipski et al., 2008), it is presented as isotropic and weak fibers onto stiff PDMS (Figure 3) that has not been observed before. Interestingly, this actin distribution is consistent with the differential cell morphology, that is, a large, circular shape onto 2.5 MPa PDMS and a small, circular shape onto softer PDMS (Figure 6). For MSCs placed onto either PDMS or glass substrate, vimentin tends to distribute over the entire cell and extends from the nucleus to the cell edge (Figure 4) as described previously (Murray et al., 2014). These clues suggested that vimentin can provide complementary support to cell stability in case of low actin expression (Figure 2C), which is consistent with previous observations that vimentin is key for protecting the cell against applied stress or stretch or to maintain cell integrity (Wang and Stamenović, 2000; Mendez et al., 2014; Chen et al., 2016). Meanwhile, this reasoning can also be used to explain why vimentin expression is upregulated dramatically on softer substratum in the current work (Figure 2D) or in the literature (Chen et al., 2016) where actin expression is low. Noticeably, vimentin is perinuclear distributed for MC3T3-E1 cells (Zhang et al., 2017) but dispersed over the entire cell for MSCs reported here, indicating the diversity of cytoskeleton remodeling in orientated substrates. Thus, future studies are required to elucidate the molecular mechanisms for these distinct distributions of actin and vimentin either for the same type of cells onto the two different substrates or for the different cell types onto the same substrate. For FAC reorganization, the normalized number or area on PDMS tends to be lower at short duration but comparable at long duration, implying the time-dependent mechanical reorganization of FACs (Figure 5). Nevertheless, the point-attached FACs are required to provide mechanical support for anchoring a cell on the substrate, and the β1 integrin–FACs–actin axis serves as one of the key mechanotransductive pathways in inducing nucleus longitudinal translocation (Figure 7).

## Conclusion

Combining both substrate stiffness and orientation helps to analyze the underlying pathways of mechanical remodeling for a cell. The density difference between the nucleus and the cytosol induces accumulatively the differential nucleus translocation for MSCs onto PDMS or glass in distinct orientations. Actin and vimentin are major components to counter-balance the nucleus translocation in either a complementary or cooperative way. The cell is stabilized mechanically onto the substrate *via* β1 integrin–FACs–actin axis.

## Data Availability

The original contributions presented in the study are included in the article/Supplementary Material, further inquiries can be directed to the corresponding author.

## Abbreviations

3D, three-dimensional; CD, cytochalasin D; CSK, cytoskeletal; ECM, extracellular matrix; FAC, focal adhesion complex; MSC, mesenchymal stem cell; PDMS, poly-dimethylsiloxane; SCs, stem cells.
