# Supplementary material for: Gravity-Vector Induces Mechanical Remodeling of rMSCs via Combined Substrate Stiffness and Orientation
Source: Front Bioeng Biotechnol. 2022 Feb 7;9:724101. doi: 10.3389/fbioe.2021.724101 (PMC8859489; doi:10.3389/fbioe.2021.724101)
Supplement: Supplementary file 1 [file DataSheet1.docx]

Supplementary Material

# Supplementary Figures


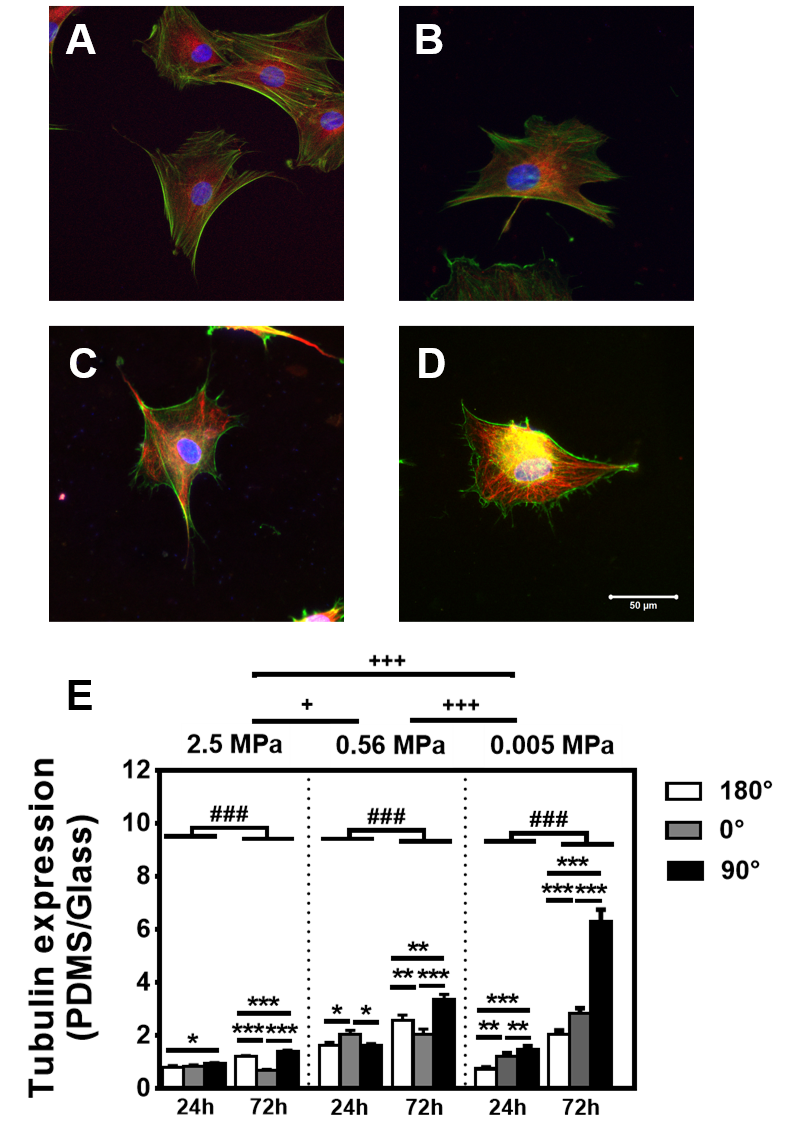


**Supplementary Figure S1.** **Comparison of tubulin expression.** (*A*-*D*) Typical images of tubulin (*red*; actin in *green* as reference) expression for cells placed onto glass (*A*, 0º, 72 h), 2.5 MPa (*B*, 180º, 24 h), 0.56 MPa (*C*, 90º, 72 h) or 0.005 MPa (*D*, 90º, 72 h) PDMS. Bar = 50 µm. (*E*) Relative tubulin fluorescent intensity onto PDMS normalized to that onto glass substrate was plotted in three orientations and presented as mean ± SE for 45 cells from three repeated experiments at 24 or 72 h. ^*^, ^**^ or ^***^, *t*-test, *p*<0.05, 0.01, or 0.001; ^###^, Two-way ANOVA test, *p*<0.001; ^+^ or ^+++^, Three-way ANOVA test, *p*<0.05 or 0.001.


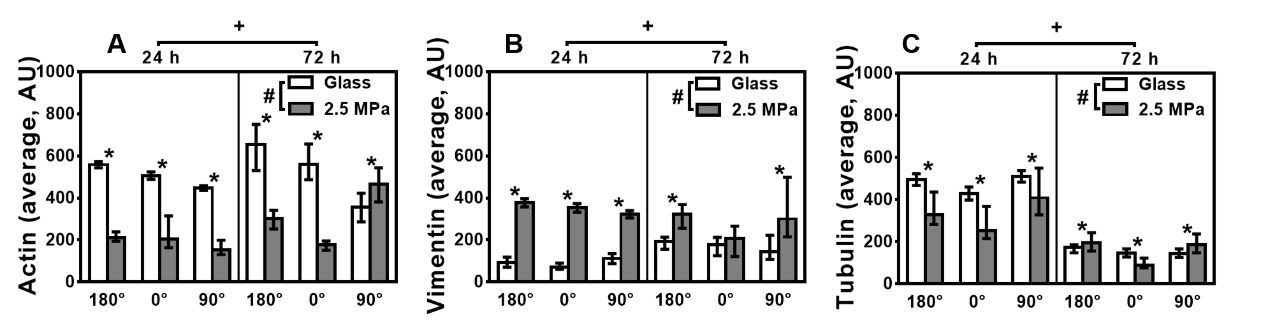


**Supplementary Figure S2.** **Expressions of cytoskeletal proteins.** Absolute fluorescent intensities for actin (*A*), vimentin (*B*), and tubulin (*C*) expression in three orientations. Data were presented as mean ± SE of 45 cells from three repeated experiments at 24 or 72 h. ^*^, *t* test, *p*<0.05; ^#^ and ^+^, Two- and Three-ANOVA tests for varied substrates and durations, respectively. *AU*, arbitrary unit.

**
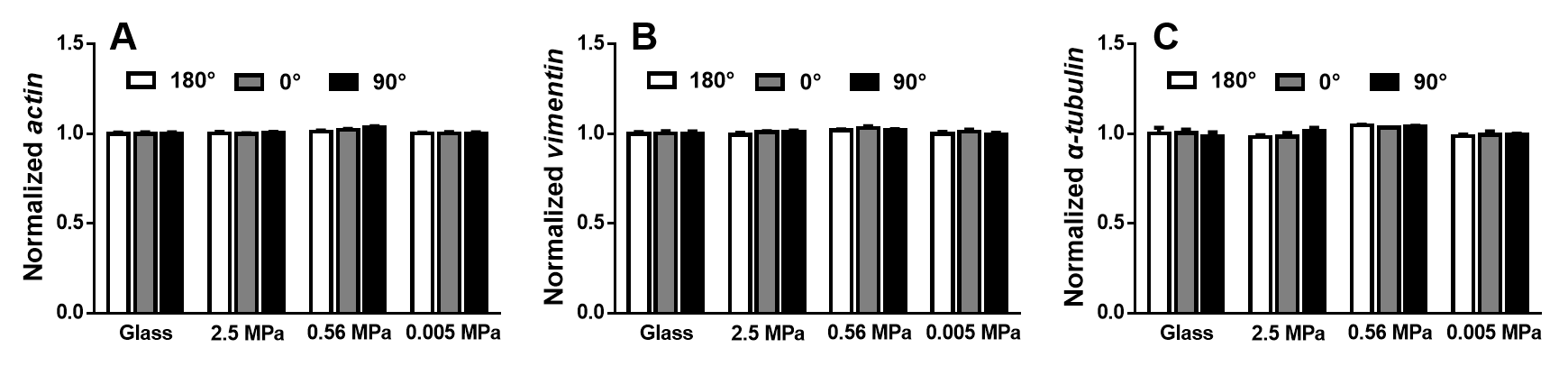
**

Supplementary Figure S3. Impacts of substrate stiffness and orientation on cytoskeleton gene expressions at 72 h. Typical mechanosensitive genes of *actin* (A)*, vimentin* (B) and *α-tubulin* (C) were analyzed by qPCR tests and their relative transcription levels were quantified from 3 repeats. Data were normalized by the one on glass at 180ºafter being calculated by their respective reference genes and presented as the mean ± SE.


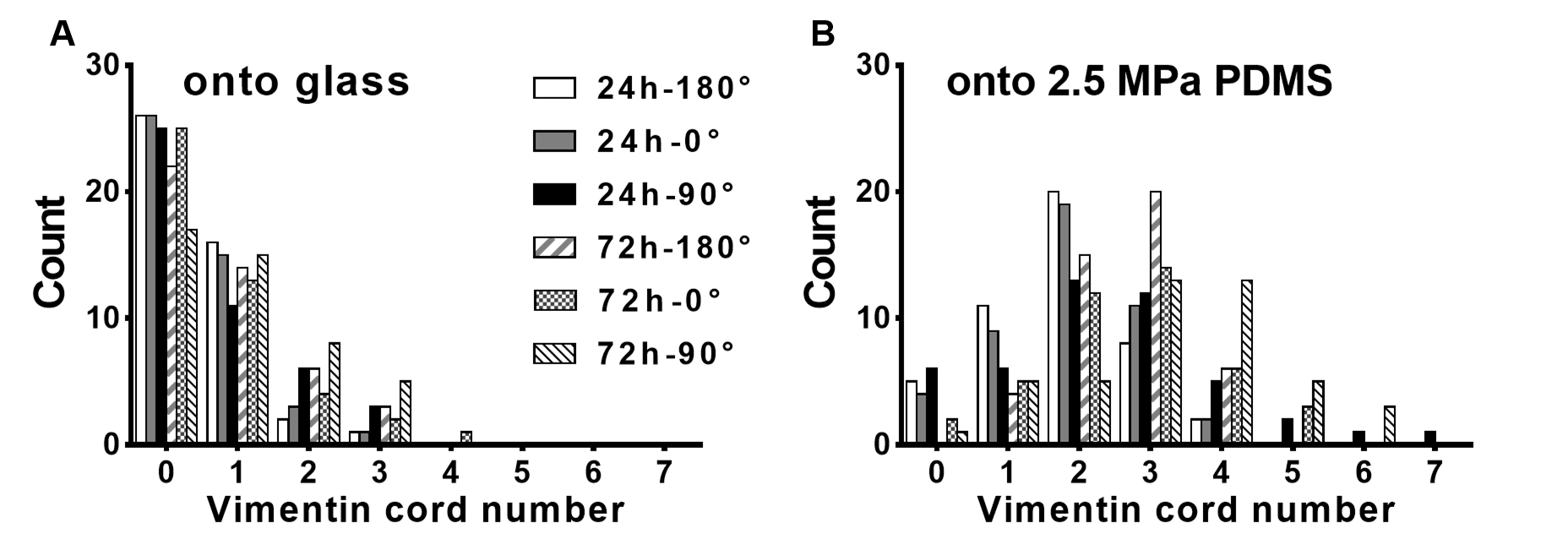


**Supplementary Figure S4. Distribution of vimentin cords.** Occurrence of vimentin cords in single cells onto glass (*A*) or 2.5 MPa PDMS (*B*) in three different orientations at 24 or 72 h. Data were collected for 45 cells from three repeated experiments.


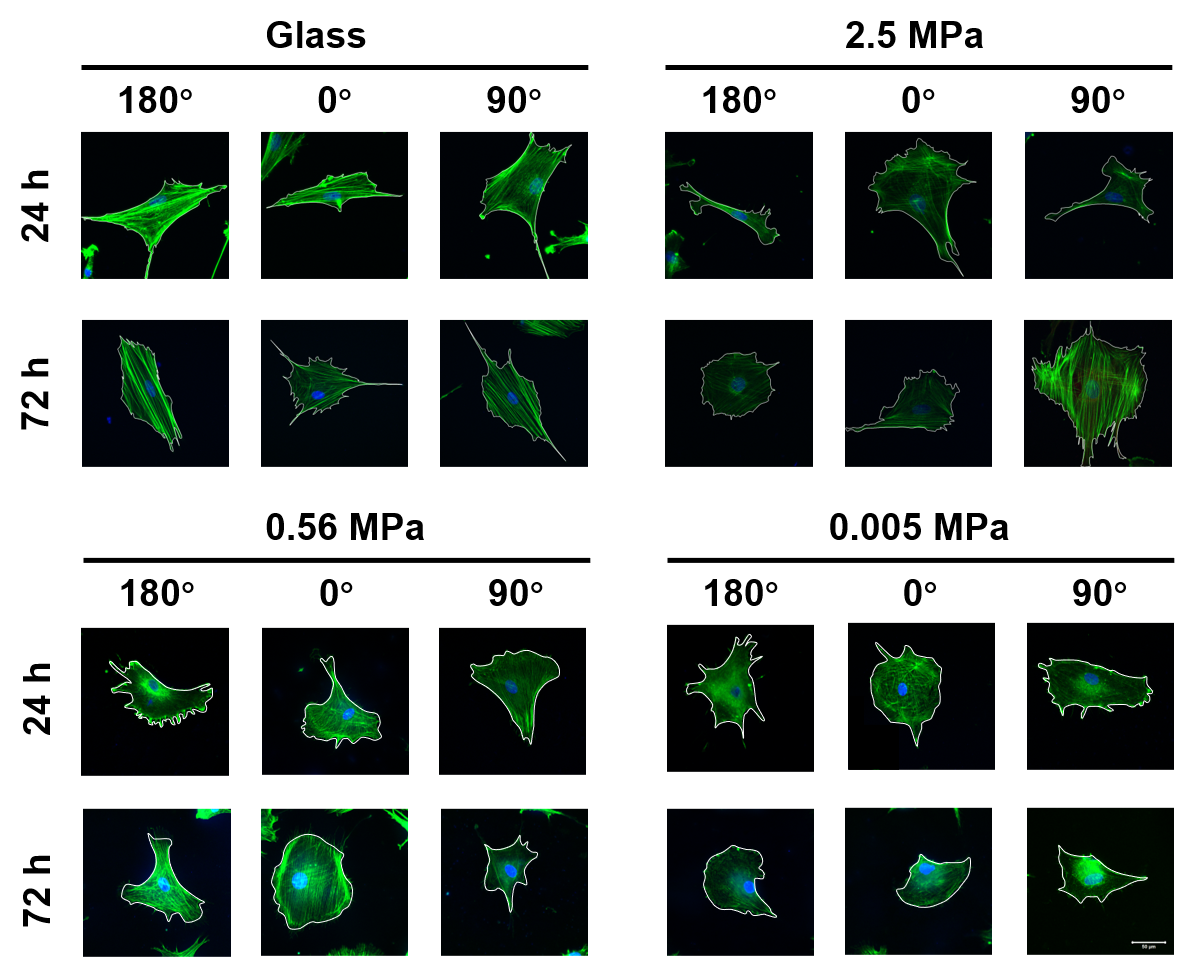


**Supplementary Figure S5. Confocal imaging of rMSCs.** Typical images used for measuring morphological parameters of cells onto glass or stiffness-varied PDMS in three orientations at 24 or 72 h. *White contour lines* were plotted upon actin (*green*) staining. Bar = 50 µm.

**Supplementary Table S1.** All the primer sequences for all the genes tested.

| **Name** | **Primer sequence** |
| --- | --- |
| *Actin* | 5’-CACGGCATTGTCACCAACTG-3’ |
|  | 5’-AACACAGCCTGGATGGCTAC-3’ |
| *Vimentin* | 5’-CAGTCACTCACCTGCGAAGT-3’ |
|  | 5’-GAGTGGGTGTCAACCAGAGG-3’ |
| *α-tubulin* | 5’-AGCGCCCAACCTACACTAAC-3’ |
|  | 5’-ATGGTAAGGCTTTCCTCAAGCA-3’ |
| *GAPDH* | 5’-GCAAGTTCAATGGCACAG-3’ |
|  | 5’-GCCAGTAGACTCCACGACA-3’ |
